# Severe, very early onset preeclampsia in a Covid 19-positive woman with a twin pregnancy presenting with a hydatidiform mole and coexisting normal fetus: a case report

**DOI:** 10.3389/fmed.2024.1340905

**Published:** 2024-02-13

**Authors:** Daniela Willy, Ralf Schmitz, Mareike Möllers, Barbara Heitplatz, Anna Kuntze, Yvonne Stratis, Katrin Bahlke, Albrecht Röpke, Matthias Meyer-Wittkopf, Kathrin Oelmeier

**Affiliations:** ^1^Department of Gynecology and Obstetrics, University Hospital Münster, Münster, Germany; ^2^Gerhard-Domagk-Institute of Pathology, University Hospital Münster, Münster, Germany; ^3^Institute of Human Genetics, University Hospital Münster, Münster, Germany; ^4^Department of Prenatal Diagnostics, Mathias-Spital Rheine, Rheine, Germany

**Keywords:** hydatidiform mole, complete mole, preeclampsia, gestational trophoblastic disease, case report

## Abstract

Cases of hydatidiform moles with a coexisting fetus are sparse and patients are at high risk for severe complications. Patients and physicians often face the dilemma of the wish to continue pregnancy until viability of the fetus while the risk for maternal complications increases. We present an educational case of a twin pregnancy presenting with a hydatidiform mole and coexisting normal fetus with a placenta praevia. The patient developed severe, early onset preeclampsia with beginning HELLP-syndrome and was tested Covid-19 positive in the further course. Termination of pregnancy was conducted via caesarean section at 18 + 6 weeks of pregnancy. Histopathology and genetic analysis confirmed a complete hydatidiform mole next to a normal placenta. Close follow-up examinations were conducted and showed normal findings including ß HCG levels normalizing within 5 months. This case combines several rare, difficult and severe medical conditions and demonstrates how an individualized therapy by an interdisciplinary team covering a highly sensitive topic was developed in a situation where no guidelines exist.

## Introduction

Gestational trophoblastic disease (GTD) is rare and treatment protocols vary among different therapeutic centers (1). GTD develops in early pregnancy. Gestational throphoblastic neoplasia (GTN) is an umbrella term that encompasses premalignant partial and complete hydatidiform moles, as well as malignant diseases such as malignant invasive moles, choriocarcinomas, placental-site trophoblastic tumors and epithelioid trophoblastic tumors (2, 3). If hydatidiform moles are not sufficiently treated, they can progress to GTN. That is why they are referred to as premalignant tumors with malignant potential (3).

GTD is rare, and cases of hydatidiform moles with a coexisting normal fetus are even less common (4). Therapeutic management is limited and often presents a huge dilemma for patients and physicians: usually there is a conflict between the parents’ wish to continue pregnancy until viability of the fetus and increasing maternal risks for development of GTN, preeclampsia, hyperthyroidism, and development of ovarian theca-lutein cysts (5, 6).

This manuscript reports the challenging clinical situation of a woman with a twin pregnancy presenting with a complete hydatidiform mole and coexisting normal fetus after a fertility treatment. The pregnancy was complicated by a placenta praevia with recurrent bleeding. In the further course, the patient developed severe preeclampsia and beginning HELLP-syndrome and was tested Covid-19 positive. This case is unique and educational as it combines several rare and difficult medical conditions and requires an individualized approach by an interdisciplinary team covering a highly sensitive topic where no guidelines exist.

## Case report

**Figure 1:**
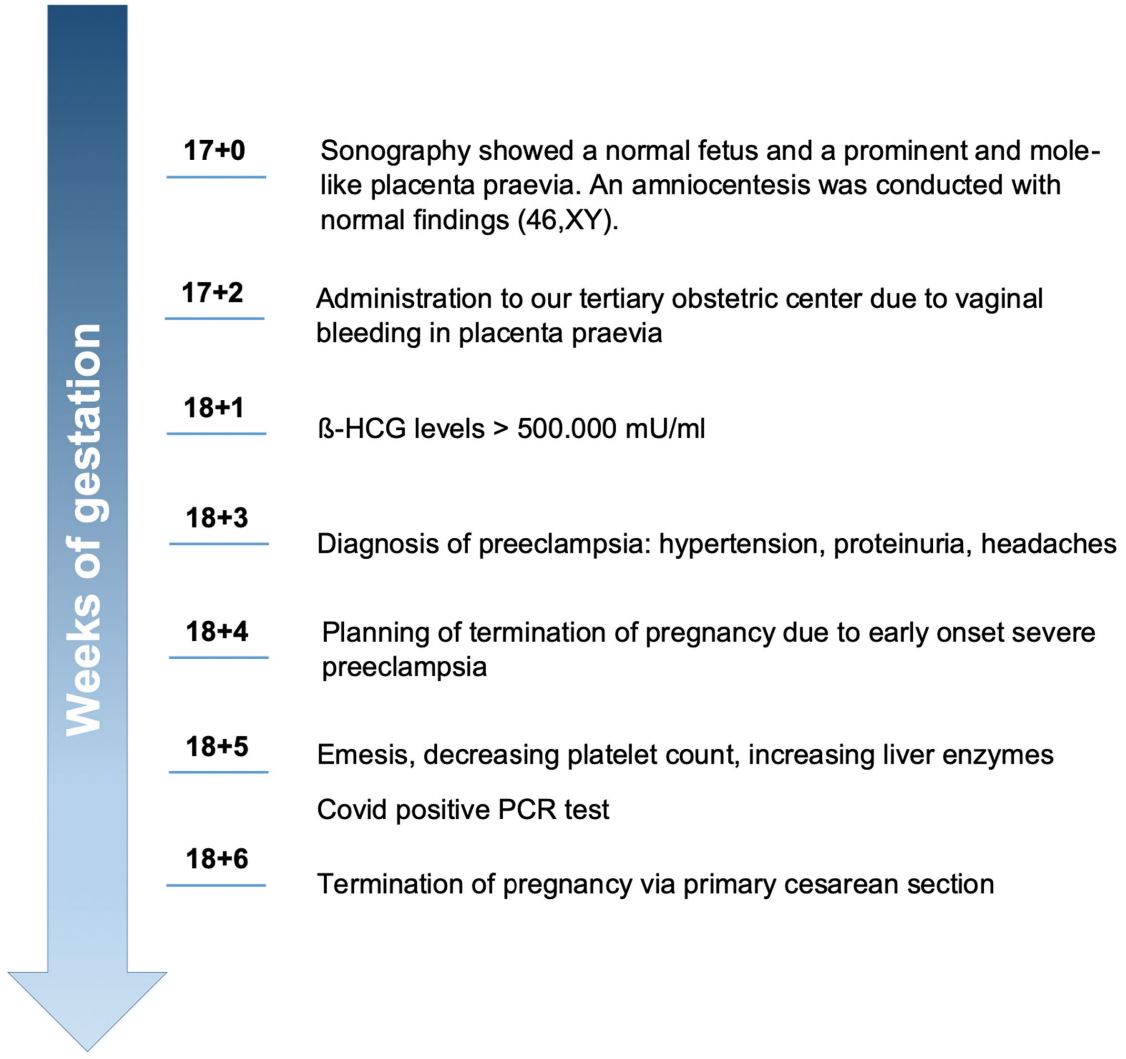
Timeline from amniocentesis to termination of pregnancy due to severe preeclampsia.

The 38-year-old primiparous Caucasian woman was referred to our hospital at 17 + 2 weeks of gestation with vaginal bleeding because of placenta praevia. Additionally, the placenta was described as mole-like. An amniocentesis had been conducted in an outpatient clinic at 17 + 0 weeks of gestation with genetic analysis showing a 46, XY karyotype as well as unremarkable results in SNP-Array. For better comprehension of the course of disease, please have a look at the timeline in Figure 1.

**Figure 2:**
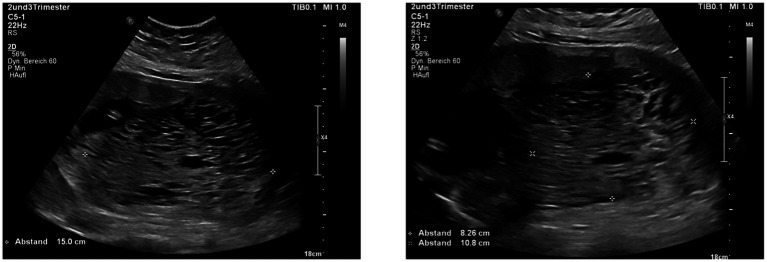
Ultrasound images showing the hydatidiform mole presenting as an enlarged placenta with numerous cysts reminding of a bunch of grapes.

The patient had experienced one abortion before and had undergone appendectomy and one cervical conisation in the past. Apart from hypothyroidism, there were no pre-existing medical conditions. She achieved pregnancy after hormonal stimulation as part of a fertility treatment. During the first trimester, no complications or specific symptoms were recorded. Upon admission to our tertiary obstetric center, vaginal bleeding was not significant. In the department of prenatal diagnostics, an ultrasound scan showed a normal fetus and an enlarged, mole-like placenta praevia, keeping with a hydatidiform mole (see Figure 2). A normal looking placenta was located at the uterine fundus, whereas a mole-like placenta was located at the lower uterine segment, covering the cervix.

In the further course, the patient developed very early onset, severe preeclampsia with hypertension, significant proteinuria (protein/creatinine ratio 1150.7 mg/g), increased liver enzymes (alanine transamninase 68 U/L, aspartate aminotransferase 57 U/L), decreased platelet count, headaches and emesis. Renal function remained normal at all times. sFlt/PlGF-ratio was increased to 162, and ß HCG levels were extremely high with >500,000 mU/mL. We recommended termination of pregnancy (TOP) in a timely manner to avoid severe maternal complications due to severe preeclampsia. Consultation and patient care were conducted interdisciplinary with specialists from several departments (Obstetrics, Neonatology, Gynecologic Oncology).

Due to the high risk for severe bleeding and the suspected hydatidiform mole, we planned a primary caesarean section via laparotomy as well as uterotomy by a longitudinal incision at 18 + 6 weeks of gestation. Shortly before surgery, the patient was tested Covid-19 positive. Primary caesarean section was conducted without complications and the patient was administrated to the Covid-19 unit afterwards. She recovered fast and was discharged from hospital one week after surgery.

**Figure 3:**
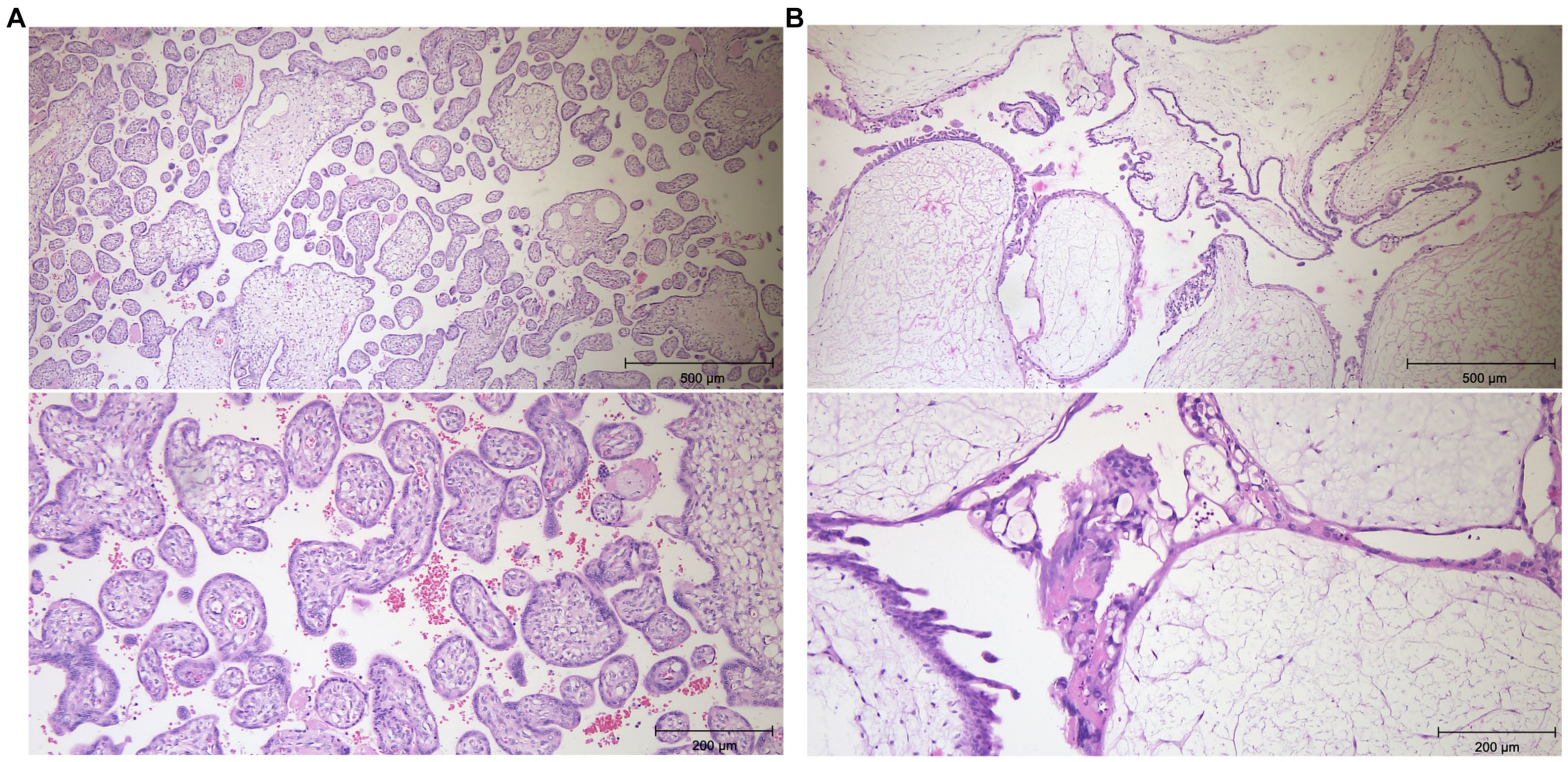
Histopathology images of the the normal placenta **(A)** and of the complete hydatidiform mole **(B)** using haematoxylin eosin staining. The pictures of the hydatitiform mole show a centrally broadened stroma with scarcely cells and pseudocystic transformation. The stroma is surrounded by a markedly proliferating accumulation of syncytio- and cytotrophoblasts. Magnification x5, x10.

Histopathology supported the suspicion of a hydatidiform mole next to a normal placenta: the complete placenta weighted 775 grams and was subdivided into two parts. One part (8.5 × 9 cm) had the appearance of a normal placenta with a matching umbilical cord and chorion. The second part was much larger (23 × 16 × 4 cm) and showed a degenerated placenta with hyperplasia of the syncytiotrophoblast, corresponding to a complete hydatidiform mole. A selection of the histopathological samples is shown in Figure 3.

**Figure 4:**
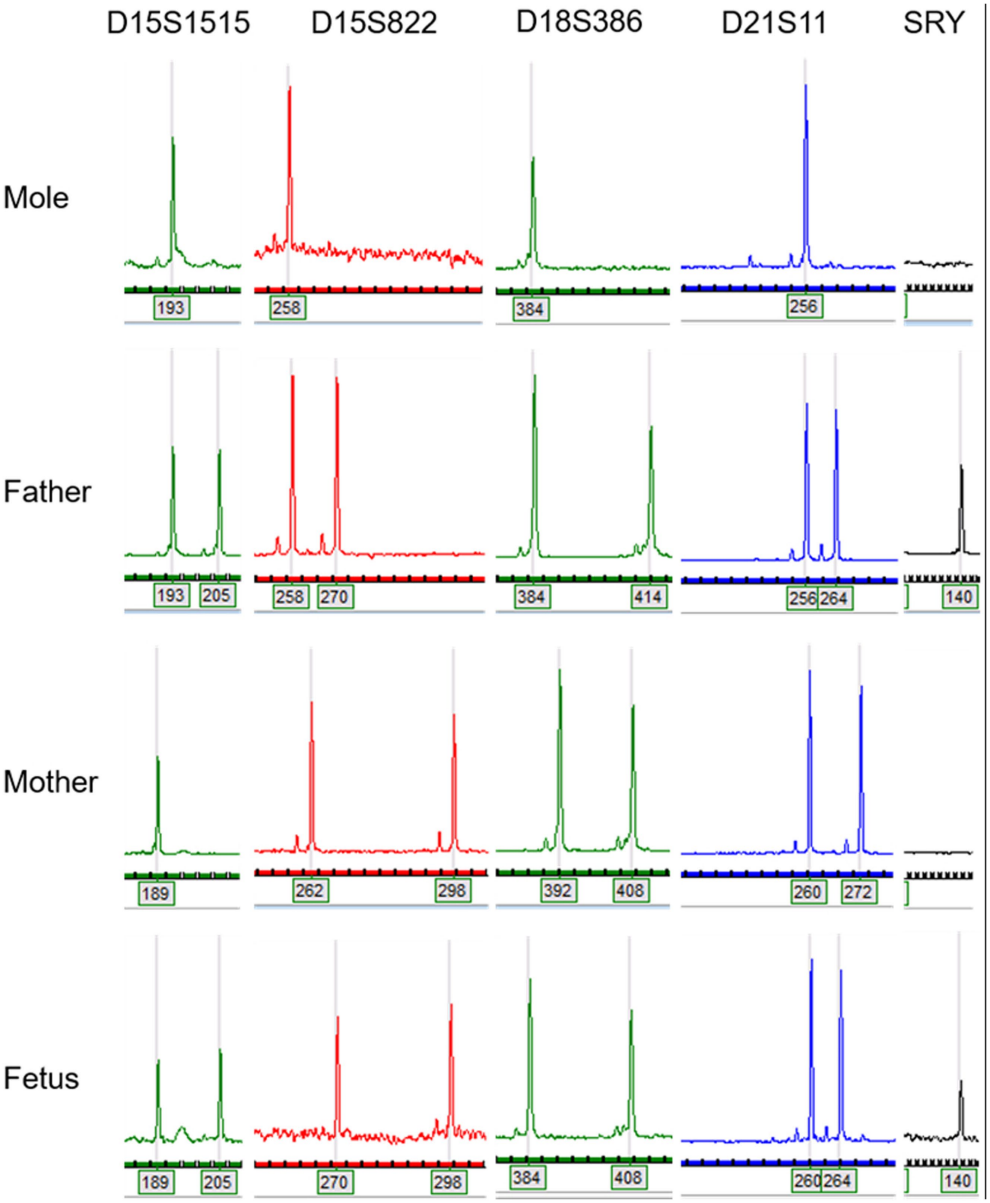
Quantitative Fluorescence PCR illustrates the absence of maternal alleles in DNA originating from hydatidiform mole. In molar DNA, only one (paternally inherited) allele could be detected for each STR- marker.

Genetic analysis (SNP-Array) on cells originating from the degenerated placental part revealed a genome-wide paternal uniparental isodisomy with a female karyotype, thus aligning with the diagnosis of a complete hydatidiform mole. In contrast, chromosomal analysis on cells originating from the normotrophic placental part revealed an unremarkable 46,XY karyotype. Hence the initial existence of a twin pregnancy consisting of a regular pregnancy with a male karyotype and a complete hydatidiform mole with a female karyotype could be confirmed. Figure 4 shows the results of Quantitative Fluorescence PCR illustrating the finding of paternal uniparental isodisomy. Since maternal mutations in genes NLRP7 and KHDC3L are associated with a genetic predisposition for recurrent hydatidiform moles, these genes were analysed in maternal blood without pathological findings.

Close gynaecological follow-up examinations were conducted and showed normal findings including ß HCG levels normalizing within 5 months. For the course of ß HCG levels, see Supplementary Figure S1.

## Discussion

This case illustrates the dilemma and challenges for patient and physicians in the rare case of a complete hydatidiform mole and coexisting normal fetus.

A limitation of this work is the limited time of follow-up and, of course, data of a future pregnancy and its outcome would be of great interest. Moreover, further information about the fetus (e.g., pathological examination of the fetus) would have been of medical interest but was not wished for by the parents.

Strengths of this study are the detailed description of this very rare medical condition, which was hampered by several additional complications, such as placenta praevia, very early onset severe preeclampsia with beginning HELLP-syndrome and Covid-19 disease, even though the latter was probably an incidental finding. Furthermore, genetic test results of amniocentesis, placenta and hydatidiform mole, and the parents are presented and give an insight into background and pathogenesis of this complex disease and help understanding it a little more.

Cases of complete hydatidiform mole and coexistent normal and viable fetuses are sparse (4). It is known that these patients are at high risk for complications, including preelampsia, GTN, persistent GTD and hyperthyroidism, while live birth-rates are low. A meta-analysis by Zilberman Sharon and colleagues points out that the incidence of maternal complications in ongoing pregnancies presenting with a hydatidiform mole and coexisting normal fetus is higher than 80% (5). Due to the rarity, no standardized procedures or guidelines exist and the diagnostic and therapeutic approach has to be planned individually by an interdisciplinary team (6–8).

Until today, there is no evaluated therapeutic option treating the hydatidiform mole while continuing pregnancy. This dilemma forces patients and physicians to make a balanced decision either to continue pregnancy in the risk of severe complications with a very close monitoring or to terminate pregnancy in order to avoid such complications. When continuing pregnancy and complications occur in the later course, it is usually recommended to terminate pregnancy immediately (4). This is what we discussed with the patient in the present case, respectively.

Moreover, the risk for intrauterine fetal growth restriction and intrauterine fetal demise seems to be high in pregnancies with hydatidiform mole and coexisting viable fetus (9, 10). The decision about continuing pregnancy, especially after assisted reproduction, is made even more difficult in the light of this evidence.

Suksai and colleagues propose to also take ß HCG levels into account, since they found evidence that when levels are <400,000 mU/mL and no maternal complications such as pregnancy-induced hypertension or hyperthyroidism develop, continuation of pregnancy could be an acceptable option and a live birth might be possible (11).

Another individual therapeutic approach is described by Al Mouallem and colleagues: a woman with a hydatidiform mole and a coexisting normal fetus without any other complications decided to receive methotrexate treatment during pregnancy to treat the hydatidiform mole. Methotrexate is known to be teratogen and is usually strictly avoided during pregnancy. The patient was able to continue pregnancy and had a live birth at 38 weeks of gestation. Tetralogy of Fallot was diagnosed in the new-born and the birth weight was 2,200 grams (12).

It also has to be taken into consideration that there is a high risk to develop GTN during pregnancy or GTN or persistent GTD postpartum (13, 14). Even the development of maternal metastasis during pregnancy is reported in literature (15). Interestingly, Lin et al. report that elective TOP does not seem to influence the risk to develop GTN (14). Another study found that 34% of all patients with complete hydatidiform mole and coexisting normal fetus were diagnosed with GTN afterwards but could not evaluate whether early elective TOP influences the risk for it (5).

Either way, patients should be monitored closely during pregnancy and afterwards. A distinct follow up postpartum including clinical examinations, ultrasound scans and ß HCG controls are mandatory in this context (4, 14).

Since the risk of recurrent molar pregnancies seems to be increased after having experienced a complete hydatidiform mole, future pregnancies should be monitored closely as well, even though maternal mutations in risk genes for recurrent hydatidiform moles were ruled out (16). Prenatal ultrasound scan in the first trimester in a specialized institution should be offered as most cases of molar pregnancy can be detected by ultrasound scan early in pregnancy (17, 18).

## Conclusion

An intensified monitoring as well as an individualized therapeutic approach are mandatory to achieve the best medical outcome possible and avoid severe and potentially life-threatening complications in patients with molar pregnancies with coexisting fetus. The prevention of those should be given the highest priority. This case illustrates that in cases of placental abnormalities even with a normal fetus, suspicion should be raised, and women should be referred to specialized centers for further diagnostics. Prenatal ultrasound scan in the first trimester can detect most cases of molar pregnancies and if a molar pregnancy is suspected, ß HCG levels should be tested. The further procedure should be an individualized and interdisciplinary approach, considering the patient’s wishes as well as potential medical risks and complications. The number of severe complications in this collective is high and a distinct follow-up postpartum is required. More data is needed to better understand this complex and challenging disease and help improve risk stratification in these patients.

## Data availability statement

The raw data supporting the conclusions of this article will be made available by the authors, without undue reservation.

## Ethics statement

Ethical approval was not required for the studies involving humans because this article is a Case Report and the patient gave informed consent. No further ethical approval was needed according to the institutional review board. The studies were conducted in accordance with the local legislation and institutional requirements. The participants provided their written informed consent to participate in this study. Written informed consent was obtained from the individual(s) for the publication of any potentially identifiable images or data included in this article.

## Author contributions

DW: Conceptualization, Data curation, Formal analysis, Investigation, Methodology, Software, Validation, Visualization, Writing – original draft, Writing – review & editing. RS: Supervision, Writing – review & editing. MM: Supervision, Writing – review & editing. BH: Data curation, Visualization, Writing – review & editing. AK: Data curation, Visualization, Writing – review & editing. YS: Data curation, Visualization, Writing – review & editing. KB: Data curation, Visualization, Writing – original draft, Writing – review & editing. AR: Supervision, Writing – review & editing. MM-W: Supervision, Writing – review & editing, Validation. KO: Conceptualization, Data curation, Formal analysis, Methodology, Supervision, Writing – original draft, Writing – review & editing.
