# Supplementary figures and images for: Severe, very early onset preeclampsia in a Covid 19-positive woman with a twin pregnancy presenting with a hydatidiform mole and coexisting normal fetus: a case report
Source: Front Med (Lausanne). 2024 Feb 13;11:1340905. doi: 10.3389/fmed.2024.1340905 (PMC10896921; doi:10.3389/fmed.2024.1340905)

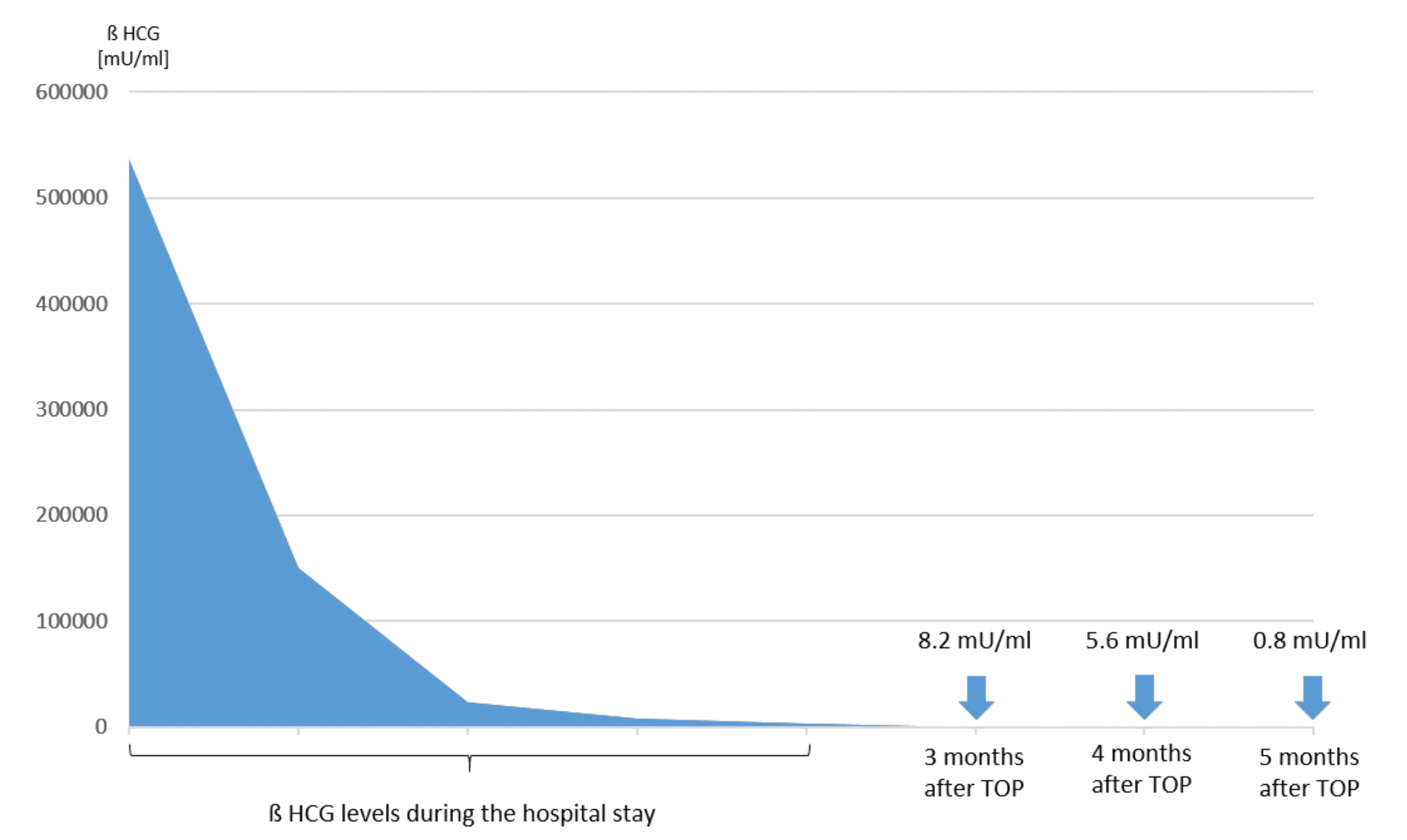

Supplement: Supplementary file 1 [file Image_1.JPEG]
